# Socio-Economic and Environmental Factors Associated with Overweight and Obesity in Children Aged 6–8 Years Living in Five Italian Cities (the MAPEC_LIFE Cohort)

**DOI:** 10.3390/ijerph13101002

**Published:** 2016-10-11

**Authors:** Tiziana Grassi, Antonella De Donno, Francesco Bagordo, Francesca Serio, Prisco Piscitelli, Elisabetta Ceretti, Claudia Zani, Gaia C. V. Viola, Milena Villarini, Massimo Moretti, Sara Levorato, Annalaura Carducci, Marco Verani, Gabriele Donzelli, Sara Bonetta, Silvia Bonetta, Elisabetta Carraro, Silvia Bonizzoni, Alberto Bonetti, Umberto Gelatti

**Affiliations:** 1 Department of Biological and Environmental Science and Technology, University of Salento, Via Prov.le Lecce-Monteroni, Lecce 73100, Italy; tiziana.grassi@unisalento.it (T.G.); francesco.bagordo@unisalento.it (F.B.); francesca.serio@unisalento.it (F.S.); 2 Euro Mediterranean Scientific Biomedical Institute, Via Reali di Bulgaria, Mesagne (BR) 72023, Italy; priscofreedom@hotmail.com; 3 Department of Medical and Surgical Specialties, Radiological Sciences and Public Health, University of Brescia, Viale Europa 11, Brescia 25123, Italy; elisabetta.ceretti1@unibs.it (E.Ce.); claudia.zani@unibs.it (C.Z.); gaia.viola@unibs.it (G.C.V.V.); umberto.gelatti@unibs.it (U.G.); 4 Department of Pharmaceutical Sciences, University of Perugia, Via del Giochetto, Perugia 06122, Italy; milena.villarini@unipg.it (M.V.); massimo.moretti@unipg.it (M.M.); sara.levorato@unipg.it (S.L.); 5 Department of Biology, University of Pisa, Via Ghini 13, Pisa 56126, Italy; annalaura.carducci@unipi.it (A.C.); marco.verani@unipi.it (M.V.); gabriele.donzelli@for.unipi.it (G.D.); 6 Department of Public Health and Pediatrics, University of Torino, Piazza Polonia 94, Torino 10126, Italy; sara.bonetta@unito.it (Sa.B.); silvia.bonetta@unito.it (Si.B.); elisabetta.carraro@unito.it (E.C.); 7 Comune di Brescia, Piazza Repubblica 1, Brescia 25100, Italy; sbonizzoni@comune.brescia.it; 8 Centro Servizi Multisettoriale e Tecnologico-CSMT Gestione S.c.a.r.l., via Branze, 45, Brescia 25123, Italy; a.bonetti@csmt.it

**Keywords:** body weight, overweight, obesity, children, socio-economic factors, MAPEC_LIFE study

## Abstract

The prevalence of obesity among Italian children has reached such alarming levels as to require detailed studies of the causes of the phenomenon. A cross-sectional study was carried out in order to assess the weight status of 1164 Italian children aged 6–8 years (the Monitoring Air Pollution Effects on Children for Supporting Public Health Policy (MAPEC_LIFE) cohort) and to identify any associations between selected socio-economic and environmental factors and overweight/obesity. The data were obtained by means of a questionnaire given to parents, and any associations were examined by binomial logistic regression analyses. Overweight was found to be positively associated with male gender, parents of non-Italian origin, and parents who smoke, and negatively associated with the parents’ level of education and employment. In addition, the frequency of overweight varied in relation to the geographical area of residence, with a greater prevalence of overweight children in the cities of central-southern Italy. This study highlights the need to implement appropriate obesity prevention programs in Italy, which should include educational measures concerning lifestyle for parents from the earliest stages of their child’s life.

## 1. Introduction

Obesity, as declared by World Health Organization (WHO) [1], is one of the most serious public health concerns in the world. It has become a global epidemic, increasing constantly in both industrialised and developing countries [2,3]. Obesity is a condition characterised by abnormal fat accumulation in adipose tissue that results in an excess of body weight and significant impairment of health, including hypertension, hyperlipidaemia, coronary heart disease, ischemic stroke, diabetes mellitus type 2, certain types of cancer, osteoporosis, and psychosocial problems [1].

The data show that excess weight affects both adults and children [4]. The first global report published in 2004 by the International Obesity Task Force (IOTF) estimated that, globally, about 155 million young people aged 5–17 years (10%) were overweight, of which 30–45 million (2%–3%) were obese [5]. Subsequently, in the WHO European Region, it was estimated that about 20% of children and adolescents were overweight, a third of whom could be classified as obese [6], while among 6–9-year-old children, 10.8% to 45.1% of boys and 15.1% to 42.3% of girls were overweight, of whom 2.8% to 14.7% of boys and 3.5% to 14.6% of girls were classified as obese [7]. More recent data [8,9] show that the prevalence of overweight in children is increasing in developing countries, but has seen a plateau or a slight decline in higher income countries. However, the problem continues to be of concern, especially in certain Mediterranean countries. Specifically, together with Spain and Greece, Italy is among the most heavily affected countries. In 2014, 30.7% of Italian children aged 8–9 were overweight, 9.8% of whom were obese, with a higher prevalence in Central and Southern regions [10].

Obesity is the consequence of a long-term imbalance between energy intake and energy expenditure, determined by food consumption and physical activity and influenced by biological and environmental factors [11]. Potential risk factors for obesity in early life include genetic factors, lifestyle, and physical, health, and environmental conditions [12,13,14,15,16,17,18,19,20], which can all in turn be influenced by the family context and socio-economic factors [19,21,22]. 

These factors affect energy balance on various levels and may interact with each other. To a certain extent, the specific causal pathways involved thus remain unclear and may differ depending on the geographical areas involved.

Given its short-term [23] and long-term consequences [23,24], as well as its relative economic burden [4,25], overweight and obesity should be prevented as early as possible. For effective intervention, it is important to identify major determinants at an early stage of life. 

In Italy, despite the presence of an effective surveillance network monitoring the weight status of children aged 8–9 throughout the country [10], there are very few studies of the factors responsible for overweight and obesity in school-age children.

The aim of this study was to: (a) assess the weight status of a cohort of Italian children aged 6–8 years based on self-reported anthropometric data; and (b) to identify any associations between selected socio-economic and environmental factors and overweight/obesity.

## 2. Materials and Methods

### 2.1. The Monitoring Air Pollution Effects on Children for Supporting Public Health Policy (MAPEC_LIFE) Project

The study cohort is the one selected for the MAPEC_LIFE project (LIFE12 ENV/IT/000614), a multicenter cohort study funded by the European Union’s LIFE+ Programme. It aims to assess the association between concentrations of certain atmospheric pollutants and early DNA damage in children aged 6–8 living in areas with varying levels of air pollution, and to build a model for the estimation of global genotoxic risk that can be used to support public health policy [26].

In order to accurately correlate biological effects with airborne pollutants, the parents of children participating in the MAPEC_LIFE study were asked to fill in a questionnaire to assess the children’s characteristics, including weight status and lifestyle, as well as any exposure factors linked to the home context that could have a confounding effect on the measured responses [27].

### 2.2. Recruitment of Children and Questionnaire Administration

The study was conducted on randomly recruited children attending primary school in five Italian cities: Brescia, Lecce, Perugia, Pisa, and Torino.

Selection of the schools and recruitment of children were conducted in accordance with a protocol [26] shared by the Research Units working in the five cities of the study: the Universities of Brescia, Salento (Lecce), Perugia, Pisa, and Torino. Classes in the first three years of eighteen schools based in 26 locations were involved. Participation in the study was voluntary, after inviting the parents of all children attending these classes. After obtaining the written consent of the parents, children meeting the following inclusion criteria were enrolled: age not below six years or above nine; residence in the cities involved in the study; absence of serious illness, absence of exposure to radiotherapy or chemotherapy in the 12 months preceding the investigation; absence of exposure to radiographic testing in the month preceding the investigation; no use of dental braces.

The data was gathered from October 2014 to March 2015 by means of a previously validated questionnaire [28] compiled by the parents. The questionnaire was composed of 148 questions, subdivided into various sections: the child’s personal information, weight and height, and health status; the characteristics of the child’s home, traffic near the school, and information on the child’s lifestyle, parents’ characteristics, and eating habits. The study cohort was composed of all children whose parents completed valid questionnaires. In total, 1164 children (3.1% of the total children aged 6–8 years living in the cities involved in the study) were enrolled.

### 2.3. Data Processing and Statistical Analysis

The data for weight and height were used to calculate the children’s body mass index (BMI), as the weight in kilograms divided by the square of the height in metres (kg/m^2^). This was used in turn to assess whether the child was underweight (UW), of normal weight (NW), overweight (OW) or obese (OB). In accordance with the indications of the IOTF, OW and OB are defined with reference to the BMI threshold values for boys and girls aged 2–18 years, calculated by Cole et al. [29] on the basis of adult values (overweight = 25 kg/m^2^; obesity = 30 kg/m^2^). The cut-off for the UW category was set at the “−2 z-score” on the basis of the BMI threshold values set out in Cole et al. [30].

Subsequently, the number of children belonging to each weight category was calculated with reference to the following personal, socio-economic, and environmental variables indicated by the parents in the questionnaires:

- Personal data: age, gender, child’s nation of birth (Italy or outside Italy), city of residence;
- Information on the parents: nation of birth (Italy or outside Italy), level of education (degree or high-school leaving certificate/lower qualification), employment status (employed or unemployed, including students and homemakers), level of employment (level I: businessman, manager, professional; level II: office worker; level III: manual worker, craftsman), smoking habits;
- Information on child’s physical activity: regular exercise (three or more times a week), outdoor sports;
- Outdoor pollution exposure factors: perceived level of traffic near the home and the school.

The data thus obtained were statistically processed using MedCalc Software version 12.3 (MedCalc Software bvba, Ostend, Belgium). For each variable, the frequency within each weight category was calculated. Binomial logistic regression analyses with the corresponding odds ratio (OR) and a 95% confidence interval (CI) were carried out to examine the possible association between personal, socioeconomic, behavioural, and environmental factors (independent variables) and excessive weight (dependent variables).

### 2.4. Ethical Aspects

The parents of all subjects gave their informed consent for inclusion before their children participated in the study. The study was conducted in accordance with the Declaration of Helsinki, and the protocol was approved by the Ethics Committee of Brescia (Project identification code 1577) on 15 January 2014. All the data were gathered and analysed in accordance with Italian Legislative Decree 196 of 30/6/2003 (“protection of personal data”) and subsequent additions, for the purposes of research. 

## 3. Results

**Figure 1:**
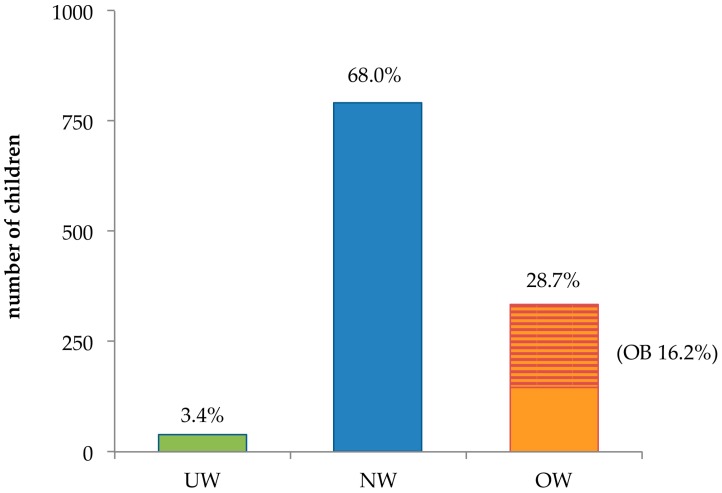
Percentage distribution in the various weight categories of children participating in the MAPEC_LIFE (Monitoring Air Pollution Effects on Children for Supporting Public Health Policy) study. UW = underweight, NW = normal weight, OW = overweight, OB = obese.

The cohort was composed of 1164 children, of whom 1111 (95.4%) were born in Italy. Regarding their weight status, 39 (3.4% of the total) were UW, 791 (68.0%) were NW, and 334 (28.7%) OW. The latter category includes 188 children who were OB (16.2%) (Figure 1).

Table 1 shows the frequencies of OW and OB children with respect to the personal variables. Regarding gender, the cohort was composed of 592 boys (50.9%) and 572 girls (49.1%). The percentage of boys in the overweight category (31.9%) was significantly higher than the girls (25.4%) (OR = 1.38, CI = 1.07–1.78). The difference was even more marked in the OB category, which accounted for 20.1% of the boys and 12.1% of the girls (OR = 1.83, CI = 1.33–2.53). 

At the moment of recruitment, 459 children (39.4%) were aged 6, 401 (34.7%) aged 7 and 304 (26.1%) aged 8. The distribution of OW and OB did not display significant differences among the various ages. 

Regarding the geographical distribution, 227 (19.5%) of the children in the cohort were resident in Torino, 250 (21.5%) in Brescia, 210 (18.0%) in Pisa, 235 (20.2%) in Perugia, and 242 (20.8%) in Lecce. In the cities of northern Italy (Torino and Brescia), there was a lower frequency of OW children (25.6%) (OR = 0.77; CI = 0.59–1.00) than those of central–southern Italy (Pisa, Perugia, and Lecce).

Table 2 shows the prevalence of OW and OB with reference to the parents’ characteristics. The parents’ nation of birth was found to be associated with children’s BMI values. Indeed, a significantly higher percentage of children with mothers born outside Italy were OW (38.9%) (OR = 1.74, CI = 1.25–2.41) than children with mothers born in Italy (26.8%). Children with fathers born outside Italy were also more likely to be OW (35.5%) (OR = 1.43; CI = 1.00–2.08) than those with fathers born in Italy (27.8%).

Regarding the parents’ level of education, 48.4% of mothers and 39.9% of fathers participating in the study have a degree. A significantly lower percentage of children with university-educated mothers are OW (24.9%) (OR = 0.70; CI = 0.54–0.90) or OB (13.9%) (OR = 0.72; CI = 0.52–0.98) than children whose mothers do not have this qualification (32.1% and 18.3%, respectively). Among children with university-educated fathers, 24.5% are OW (OR = 0.72; CI = 0.55–0.94) and 12.0% are OB (OR = 0.59; CI = 0.42–0.83). 

The prevalence of overweight and obesity in the children included in the cohort do not seem to be correlated with the parents’ rate of employment. In contrast, regarding the type of employment, it emerged that among children whose mothers have high-level employment (businesswoman, manager, professional) 23.9% are OW (OR = 0.67; CI = 0.48–0.93) and 9.9% are OB (OR = 0.46; CI = 0.30–0.72). At lower levels of maternal employment (office workers, manual workers, and craftswomen) the percentage of OW and OB is significantly higher. Children are also less likely to be OW (23.7%; OR = 0.64; CI = 0.49–0.85) and OB (12.2%; OR = 0.58; CI = 0.41–0.83) when their fathers are employed at a high level.

Parents who smoke were correlated with excess body mass in children. Specifically, 33.9% of children with mothers who smoke were OW (OR = 1.36; CI = 1.00–1.86), including 24.2% OB (OR = 1.93; CI = 1.36–2.75), while 32.2% of children with fathers who smoke were OW (OR = 1.28; CI = 0.97–1.70), including 21.7% OB (OR = 1.72; CI = 1.24–2.39).

Table 3 shows the distribution of OW and OB with respect to the level of motor traffic perceived by the parents near the home and the children’s school. The data do not seem to indicate significant differences in weight between children living in areas with heavy traffic and those living in areas with light or moderate traffic.

Of the children participating in the study, 44.8% regularly exercise three or more times a week, in 28.3% of cases in the open air (Table 4). The variables linked to the children’s physical exercise patterns were not found to be significantly correlated with their weight status.

## 4. Discussion

The data on the anthropometric variables of 1164 subjects included in the MAPEC_LIFE cohort made it possible to identify children with different body masses and to assess their distribution in the various weight categories (UW, NW, and OW, the latter including OB) on the basis of the criteria indicated by the IOTF [29,30]. The detected frequencies of children with excess weight appear to be comparable with data from other studies carried out in the WHO European Region [7,31,32] and confirm the relatively high and worrying proportion of OW and OB among Italian children. In line with other studies [10,33,34] conducted in Italy, the data also highlighted the existence of a north-south gradient regarding the frequency of overweight children, which was found to be lower in the cities of the north (Torino and Brescia) and higher in those of the centre and south (Pisa, Perugia and Lecce). Although this trend has been extensively described, it has not been clearly explained. It is probably linked to social, economic, and cultural differences between the northern and southern regions of Italy [35].

Indeed, this study showed that the weight status of the children was closely associated with personal, socio-economic, and environmental factors investigated by means of questionnaires filled in by the parents.

One of these factors is gender. In our study, a significantly greater percentage of boys than girls were OW and OB. However, this pattern is not clearly confirmed by the literature. Specifically, in studies of Italian children based on IOTF cut points [10,36], the association between male gender and excess body weight is very weak or absent. In this case, the observed phenomenon may again be linked to social and cultural factors, but it may also be the result of an artifice arising from confounding factors, which means that a more detailed investigation is required in order to confirm and justify this association. 

The socio-economic factors considered in the study of the prevalence of OW and OB among children were: the parents’ nation of birth, level of education, employment status, and type of occupation.

Children with parents born outside Italy were more likely to be OW and OB than children with parents born in Italy. This observation is in line with the results of other studies conducted in the United Kingdom [37], Luxembourg [21], and Germany [19], in which this association is explained with reference to the subjects’ ethnicity, the socio-economic level of families with an immigrant background, and the continuation of certain “obesogenic” habits and behaviours. Additionally, in our study, these factors could explain the high prevalence of OW among children with parents born outside Italy. Further studies may be conducted in order to determine any differences in the distribution of OW depending on the countries of origin.

The impact of social position on the risk of obesity in developed countries has been discussed by several authors [22,32,38,39,40]. They observe that among the various measures used to classify social position, parental education has the most consistent association with OW and obesity in childhood: the lower the level of parental education, the greater the probability that children are OW or OB. It is likely that less well-educated parents also have less knowledge of healthy eating, but other factors linked to low education level may contribute to the creation of an “obesogenic” environment. Our study also found a negative correlation between the parents’ level of education and the child’s body mass. 

While the parents’ employment status (employed or unemployed) does not seem to influence the weight of children in the MAPEC_LIFE cohort, their level of employment could be associated with BMI. A high professional level of parental employment—particularly of the mother—seems to have a protective effect on the children regarding the risk of obesity. A high level of employment is often correlated with a high level of education as well as a greater level of socio-economic well-being, which also entails access to goods and services (gymnasia, swimming pools, and other structures specialising in physical well-being, domestic help, etc.), which can create a protective environment in terms of various health problems, including obesity [19,41,42]. In addition, dieting and healthy weight-control practices such as reducing high energy food and fat intake and a high level of exercise are more common in women of a higher socio-economic status, who may transfer such behaviours to their children [22,43].

Additional findings of our study include the association between parental smoking—with particular reference to maternal smoking—and filial overweight. These findings are supported by similar studies conducted in very different social and cultural settings [38,44,45], in which parental smoking was found to be an independent risk factor for obesity in children, suggesting the causality of this association [38]. However, the underlying mechanism responsible for the association is still unclear. Some authors have suggested that the relationship may be linked to the smoking habits of parents (especially the mother) during pregnancy, the poor eating habits of smoking parents, or additional risk-related behaviours which make their children more inclined to become overweight and obese.

Despite the evidence presented in the literature regarding the association between traffic density, traffic-related air pollution, and the development of obesity in children [20], in our study no significant relationship was seen. It should be considered, however, that the data on traffic levels used in our analysis were based on parents’ perceptions and were therefore influenced by subjective bias that is hard to quantify.

The data on physical exercise also do not seem to be associated with overweight. In this case, the transversal data gathering could have neglected the children’s previous behaviours, considering only the exercise habits at the time of the interview, which for some children may themselves be a response to overweight or obesity.

The data obtained in the present study confirm the important role of factors linked to the family context in determining the excess body mass of children in the MAPEC_LIFE cohort. Children’s lifestyles depend almost entirely on the environment that the parents create around them from gestation onwards [13,15,16]. Previous research has shown that quantitative and qualitative aspects of the child’s diet and exercise patterns are considerably influenced by the family environment [14,18], and that the family plays an important role in both the development and prevention of weight problems in children [11,19,21]. As well as being closely linked to socio-economic status, this environment largely depends on the parents’ knowledge of factors affecting health and disease [22,38]. For this reason, obesity prevention programmes need to include effective educational provisions for both parents and children. In addition—as has been described in a pilot study conducted in the framework of this same project [46]—children have been proven to possess great sensitivity to these themes and to be fast learners regarding the concepts and lifestyles that prevent adverse health effects. 

## 5. Conclusions

The results of this study provide information on the variables associated with the weight status of a group of 1164 children aged 6–8 resident in five Italian cities. Gender and socio-economic factors such as the parents’ nationality, level of education, and level of employment, as well as whether the parents are smokers, were found to be closely associated with overweight/obesity among the children. The distribution of subjects’ weight status also highlighted differences linked to the geographical area of residence, with a greater prevalence of overweight children in the cities of the centre and south of Italy. This study highlights the need to implement appropriate obesity prevention programs in Italy, which should include educational measures concerning lifestyles for parents from the earliest stages of their children’s lives. 

This study is not without its limitations. The anthropometric data used in this work to calculate BMI and body size were self-reported by the parents who compiled the questionnaires, and were not measured in accordance with standardised methods. In addition, only some of the factors potentially affecting the children’s weight status were considered, and only basic analyses were carried out. Further studies could include the analysis of genetic factors, the family history of certain diseases such as obesity, the objective measurement of environmental parameters, the previous lifestyles and eating habits of the children and their parents (rather than just at the time of the interview), and analysis of the data by multinominal logistic regression, which can take all possible factors and their individual weight into account when determining overweight and obesity.

## Figures and Tables

**Table 1:** Characteristics of the children in the MAPEC_LIFE (Monitoring Air Pollution Effects on Children for Supporting Public Health Policy) cohort.

Variable		(*n*)	OW (Including OB)		OB	
			%	OR (CI)	%	OR (CI)
Gender	Boys	(592)	31.9	1.38 (1.07–1.78)	20.1	1.83 (1.33–2.53)
	Girls	(572)	25.4	-	12.1	
Age	6 years	(459)	29.6	1.08 (0.83–1.40)	17.0	1.11 (0.81–1.52)
	7 years	(401)	26.9	-	15.2	-
	8 years	(304)	29.6	-	16.1	-
Area of residence	North	(477)	25.6	0.77 (0.59–1.00)	14.5	0.81 (0.59–1.11)
	Centre–South	(687)	30.9	-	17.3	-

OW = overweight, OB = obese, OR = Odds Ratio, CI = 95% Confidence Interval.

**Table 2:** Characteristics of the parents of the children in the MAPEC_LIFE cohort.

Variable		(*n*)	OW (Including OB)		OB	
			%	OR (CI)	%	OR (CI)
Mother’s Nation of Birth	Outside Italy	(180)	38.9	1.74 (1.25–2.41)	20.6	1.43 (0.96–2.13)
	Italy	(984)	26.8	-	15.3	-
Father’s Nation of Birth	Outside Italy	(138)	35.5	1.43 (1.00–2.08)	20.3	1.38 (0.88–2.15)
	Italy	(1026)	27.8	-	15.6	-
Mother’s Level of Education	University Degree	(563)	24.9	0.70 (0.54–0.90)	13.9	0.72 (0.52–0.98)
	High School or Lower	(600)	32.1	-	18.3	-
Father’s Level of Education	University Degree	(457)	24.5	0.72 (0.55–0.94)	12.0	0.59 (0.42–0.83)
	High School or Lower	(689)	31.0	-	18.7	-
Mother Employed	Yes	(853)	29.3	1.13 (0.85–1.52)	16.3	1.04 (0.73–1.48)
	No	(310)	26.8	-	15.8	-
Father Employed	Yes	(1022)	28.7	1.16 (0.76–1.77)	16.2	1.14 (0.68–1.92)
	No	(124)	25.8	-	14.5	-
Mother’s Occupation	Level I ^1^	(272)	23.9	0.67 (0.48–0.93)	9.9	0.46 (0.30–0.72)
	Level II ^2^	(389)	30.1	-	18.5	-
	Level III ^3^	(192)	35.4	-	20.8	-
Father’s Occupation	Level I ^1^	(427)	23.7	0.64 (0.49–0.85)	12.2	0.58 (0.41–0.83)
	Level II ^2^	(279)	32.3	-	19.0	-
	Level III ^3^	(315)	32.7	-	19.4	-
Mother Smoker	Yes	(227)	33.9	1.36 (1.00–1.86)	24.2	1.93 (1.36–2.75)
	No	(936)	27.4	-	14.2	-
Father Smoker	Yes	(323)	32.2	1.28 (0.97–1.70)	21.7	1.72 (1.24–2.39)
	No	(822)	27.0	-	13.9	-

^1^ businesswoman/businessman, manager, professional; ^2^ office worker; ^3^ manual worker, craftswoman/craftsman.

**Table 3:** Level of traffic perceived by parents near the home and the children’s school in the MAPEC_LIFE cohort.

Variable		(*n*)	OW (Including OB)		OB	
			%	OR (CI)	%	OR (CI)
Level of Traffic near the Home	Heavy	(460)	27.6	0.92 (0.71–1.19)	15.7	0.94 (0.68–1.30)
	Moderate or Light	(704)	29.4	-	16.5	-
Level of Traffic near the School	Heavy	(603)	29.5	1.09 (0.84–1.40)	17.1	1.15 (0.84–1.58)
	Moderate or Light	(561)	27.8	-	15.2	-

**Table 4:** Exercise/sport practised by the children in the MAPEC_LIFE cohort.

Variable		(*n*)	OW (Including OB)		OB	
			%	OR (CI)	%	OR (CI)
Sport (≥3 Times/Week)	Yes	(522)	31.0	1.23 (0.95–1.59)	18.2	1.31 (0.96–1.79)
	No	(642)	26.8	-	14.5	-
Outdoor Sports	Yes	(329)	28.3	0.97 (0.73–1.29)	16.1	1.00 (0.70–1.41)
	No	(835)	28.9	-	16.2	-
